# Burnout and Cognitive Performance

**DOI:** 10.3390/ijerph18042145

**Published:** 2021-02-22

**Authors:** Panagiota Koutsimani, Anthony Montgomery, Elvira Masoura, Efharis Panagopoulou

**Affiliations:** 1 Department of Educational & Social Policy, School of Social Sciences, Humanities and Arts, University of Macedonia, Egnatia 156, 54636 Thessaloniki, Greece; monty5429@hotmail.com; 2 Department of Experimental Cognitive Psychology, School of Psychology, Aristotle University of Thessaloniki, 54124 Thessaloniki, Greece; emasoura@psy.auth.gr; 3 Laboratory of Hygiene, Medical School, Aristotle University of Thessaloniki, 54124 Thessaloniki, Greece; efharis@gmail.com

**Keywords:** burnout, cognitive functioning, depression, anxiety, family support

## Abstract

The aim of this study was to investigate the relationship between burnout and cognitive functioning. The associations of depression, anxiety and family support with burnout and cognitive functioning were also examined both independently and as potential moderators of the burnout–cognitive functioning relationship. Seven different cognitive tasks were administered to employees of the general working population and five cognitive domains were assessed; i.e., executive functions, working memory, memory (episodic, visuospatial, prospective), attention/speed of processing and visuospatial abilities. Burnout, depression, anxiety and family support were assessed with the Maslach Burnout Inventory-General Survey, the Hospital Anxiety and Depression Scale and the Family Support Scale respectively. In congruence with the first and fourth (partially) Hypotheses, burnout and perceived family support are significantly associated with some aspects of cognitive functioning. Moreover, in line with the third Hypothesis, perceived family support is inversely related to burnout. However, in contrast to the second and fourth Hypotheses, depression, anxiety and perceived family support do not moderate the burnout–cognitive functioning relationship. Additional results reveal positive associations between burnout depression and anxiety. Overall findings suggest that cognitive deficits, depression and anxiety appear to be common in burnout while they underpin the role of perceived family support in both mental health and cognitive functioning. Implications for practice are discussed.

## 1. Introduction

People who suffer from burnout tend to complain about cognitive deficits. Particularly, these individuals often report reduced problem—solving and learning abilities and difficulties in staying focused during daily tasks, and failing to keep important information such as names or appointments [1,2,3], observations that are also replicated in more recent studies [4,5,6,7,8]. Interestingly, this subjective mental fatigue appears to persist after three years of the diagnosis [9], a finding which indicates that subjective cognitive deficiencies might have long-lasting consequences in the everyday functioning of burnout individuals. However, apart from the subjective complaints, employees suffering from burnout also exhibit cognitive deficits even when measured with objective neuropsychological batteries.

## 2. Burnout and Cognitive Functioning

Cognitive functioning has started receiving more attention in burnout research during the past years. In fact, burnout has been found to be associated with cognitive impairment with the predominant deficits concerning executive functions, attention and memory [10]. Executive functions (or executive control) are an important cognitive domain responsible for the coordination and regulation of our thoughts and behavior towards chosen goals [11,12]. Executive control deficits among individuals with burnout were observed in the study of van Der Linden et al. [13], who found that burnout is associated with poor performance in tasks of sustained attention and inhibition, the two important components of executive control; the third dimension of executive functioning is switching (or flexibility), i.e., the ability to shift between multiple tasks [14]. Similarly, in another study exhaustion in teachers was negatively related to cognitive performance with the suggestion that deficits in executive control are a potential mechanism to explain the impaired cognitive performance [5].

Other researchers showed that people who were diagnosed sometime in the past with work–stress-related exhaustion performed worse on cognitive tasks requiring attention and visuo-spatial constructional ability than healthy employees [8]. Moreover, Jonsdottir et al. [15] found that burnout patients perform poorer compared to healthy controls on executive control tasks, attention span, working memory, learning and episodic memory. Mild differences in neuropsychological screening were also found in the study of Eskildsen et al. [16], where patients with work-related stress performed worse compared to healthy employees. Follow-up studies showed that some aspects of cognitive functions were still impaired even after certain follow-up periods [4,17,18,19]. Notably, even motivational interventions do not appear to be beneficial in strengthening the cognitive performance of burnout individuals [19,20].

Considering the fact that burnout is the result of chronic occupational stress [21], the observed cognitive deficits among burnout employees are not unexpected. Chronic stress can have a long-term negative impact on the brain. Glucocorticoids (GCs), a class of hormones released upon exposure to stressful situations [22], have access to brain regions, such as the hippocampus, amygdala and frontal lobes, and when stress becomes prolonged this can be destructive of the neurons in these brain areas [23]. Thus chronic exposure to stress can have negative effects on both cognition and the onset of various psychological syndromes such as burnout, depression, and anxiety [24]. Indeed, Durning et al. [25] found that high burnout depersonalization scores were associated with a decreased blood oxygenation level dependent (BOLD) effect in the right dorsolateral prefrontal cortex (dlPFC) and middle frontal gyrus, while high exhaustion scores were associated more with a right posterior cingulate cortex and middle frontal gyrus BOLD signal [25], three brain regions that are associated with executive functions [26,27], memory and attention, respectively [28,29]. Similarly, Blix et al. [30] found that individuals who scored high in burnout exhibited significant reductions in the grey matter of the anterior cingulate cortex and the dlPFC in caudate and putamen volumes.

Hence, and given the fact that exposure to chronic and uncontrollable stress can disrupt the prefrontal cortex–dependent processes [31,32] and reduce the activation of the dlPFC [33,34], cognitive deficits might be also present in burnout individuals. Research has shown that higher burnout scores are correlated with increased brain activation when performing tasks requiring attention, indicating that burnout individuals are using more brain resources in order to complete these cognitive tasks [35]. The pattern of neurological deficits that appear to be affected most in burnout individuals are mainly related to changes in the frontal brain regions rather than hippocampal structures [34,36], brain areas that are mainly responsible for high-order cognitive functions that could intervene with the individuals’ everyday cognitive functioning.

However, not all studies have observed cognitive deficits among burned out employees. Österberg et al. [37], even though they found that burnout individuals performed slightly worse in a task of perceptual speed, did not find any other significant differences compared to healthy employees in the other cognitive domains assessed. Similarly, McInerney et al. [38] did not find any significant relationships between neurocognitive performance and burnout in a group of psychiatric nurses. An unexpected finding was observed in the study of Castaneda et al. [39] who found that employees who reported high burnout levels showed better verbal working memory skills and higher general intelligence compared to participants with lower burnout levels. Working memory is a part of executive functioning and is an important mechanism for performing everyday tasks such as reading [40], comprehension [41] and decision making [42]. Burnout is common among goal-oriented [43] individuals while higher executive functions can protect an individual from negative stress effects [44]. Thus, one cannot rule out the possibility that these individual differences can underly the inconsistencies among studies. Moreover, in their study Castaneda et al. [39] examined non-clinical employees, thus the participants where possibly at the initial burnout stages, suggesting the positive effects of acute stress in immediate memory tasks [45]. Indeed, cognitive deficits have been mainly observed among clinical burnout employees suggesting the existence of different cognitive patterns depending on the burnout phase. In their study, Oosterholt et al. [18] show aptly this differentiation, as they studied three different groups (clinical burnout, non-clinical burnout and healthy group) and they found that only the clinical burnout group had significantly, but marginally, lower cognitive performance in only one (out of five) cognitive tasks, while the healthy and the non-clinical burnout group exhibited similar cognitive abilities.

Another reason for the above inconsistencies could be differentiation among the tools being used to assess cognitive functions. Moreover, another important factor that can provide misleading observations is that burnout might indirectly affect cognitive performance via other underlying mechanisms. Depression and anxiety often co-exist with burnout while they have also been found to be linked with cognitive deterioration. Furthermore, non-work related factors such as high perceived social and family support have been associated with lower burnout levels [46] and better cognitive functions [47], possibly explaining the surprising findings of Castaneda et al. [39]. Nevertheless, not all studies examining the burnout–cognitive functioning relationship assess for these confounding factors that could underlie the relationship.

## 3. Burnout, Depression, Anxiety and Cognitive Functioning

Although burnout is a chronic work-related condition, it often co-exists with depression and anxiety, often making it impossible to distinguish from these two disorders [48]. In fact, there is a debate among researchers on the exact relationship between burnout and depression as some scholars regard burnout as a depression dimension [49] while others consider the two as different constructs [48]. With respect to the burnout–anxiety relationship, although less commonly investigated, these two psychological phenomena show strong and positive associations [50,51]. However, in a recent meta-analysis it was shown that burnout is differentiated from both depression and anxiety as studies that observe significant associations show mainly medium effect sizes while the tools being used to measure for these three phenomena also appear to affect the observed associations [48].

Apart from their similarities regarding mental health, burnout, depression and anxiety also appear to share similar cognitive patterns. Interestingly, both depression and anxiety can have negative effects on memory [52,53,54], executive functions [52], attentional control [55] and even increased risk of dementia onset [56], cognitive deficits also observed in burnout. Hence, one could argue that these two psychological syndromes could moderate the relationship between burnout and cognitive functioning [21]. That is, the observed cognitive deficits among burned-out employees could be accentuated by depression and/or anxiety and, thus, not reflect a burnout consequence per se. Therefore, in this study we aimed to measure for depression and anxiety feelings as possible moderators in the relationship between burnout and cognitive functioning.

## 4. Burnout and Family Support

Considering the fact that in today’ society with the rise of technology (e.g., laptops) which allows people to work from home, the distinction between work and home life has become blurred [57]. Several studies have shown that family support, i.e., having someone at home you can talk to and is able to help you, is related to low burnout levels [46], even when job demands increase [58]. However, the relationship between family network and workers’ mental health is still inconclusive and further investigation is needed [59,60]. As typical Greek families are characterized by close-knit relationships [61], we examined if family support can have an influence, either by enhancing or by protecting an individual from developing burnout. As mentioned above, family support can have beneficial effects in cognitive performance. Therefore, an additional goal of this study was to examine its effects on cognitive performance both directly and indirectly as a moderator between burnout and cognitive performance. By conducting this moderation analysis, we aimed to explore if potential burnout consequences in cognitive performance are alleviated by the perceived family support.

## 5. Objectives and Hypotheses of the Study

The main objective of the present study was to investigate the relationship between burnout and cognitive functioning. An additional aim was to examine whether depression, anxiety and perceived family support are associated with cognitive performance and if they moderate the burnout–cognitive functioning relationship. A third goal was to explore for any associations among burnout, depression, anxiety and perceived family support.

Our primary hypothesis is that burnout is negatively related with cognitive performance (as measured by administered cognitive tests). Our secondary hypothesis is that depression and/or anxiety moderates the relationship between burnout and cognitive performance by enhancing the observed association. Our third hypothesis is that perceived family support is inversely related to burnout levels. Our fourth hypothesis is that greater family support will positively affect cognitive performance (and vice versa) while it will moderate the burnout–cognitive functioning relationship by reducing the effects of burnout on cognitive functioning.

## 6. Materials and Methods

### 6.1. Ethics

The authors’ university does not have an Institutional Review Board (IRB); therefore, it was not possible to get IRB approval for the research. Hence, we followed the appropriate ethical procedures of the declaration of Helsinki when conducting research with human subjects. All participants were given a written informed consent form for voluntary participation prior to their participation.

### 6.2. Procedure and Participants

The present study is of a cross-sectional design and relationships among burnout, depression, anxiety, perceived family support and cognitive performance were examined. All studied variables were continuous, thus their values ranged from low to high. The examination sessions took place either at the researchers’ university or the participant’s workplace. Since all participants were actively working, the assessments were conducted at their time of convenience; either in the morning or in the evening. As participants arrived, they were given an informed consent form which stated the aims and details of the study, its voluntary nature and that they can cease participation at any time. Participants were also asked whether they had any questions about the study and then asked to sign the informed consent form. After obtaining the informed consent, they were asked about certain demographic features. Then the researcher began the cognitive evaluation and after its completion the participants were given the three self-reported questionnaires to fill in. At the end of the assessment, the participants were asked again if they had any questions and if they wanted to be e-mailed with the results of their assessment, and were thanked for their cooperation.

A total of 104 employees were recruited from the general working population. The sampling method we used was snowball sampling, a non-probability sampling method which is widely used across the social sciences and acts as an auxiliary means for accessing participants and enriching the sample size [62]. In the present study we contacted employees from our social and academic environment; after the completion of the assessment some participants informed us that they were also able to recruit other participants for the study.

### 6.3. Assessment Tools

#### 6.3.1. Burnout

We administered the Maslach Burnout Inventory-General Survey (MBI-GS) [63] for evaluating burnout levels. The MBI-GS consists of a Likert-type self-reported questionnaire which consists of 16 items and is designed to assess the three components of burnout, i.e., exhaustion, cynicism and personal efficacy [63]. Higher scores on exhaustion and cynicism and lower scores on personal efficacy suggest higher burnout levels. Cronbach’s alphas for the three subscales were α = 0.90 for exhaustion; α = 0.71 for cynicism; and α = 0.84 for personal efficacy.

#### 6.3.2. Depression and Anxiety

The Hospital Anxiety and Depression Scale (HADS) [64] was administered for measuring anxiety and depression feelings. Particularly, HADS is a brief self-administered scale which consists of 14 items scored as two 7-item subscales measuring anxiety and depression [65]. Although it was originally developed for use in hospital patients, now it is widely used across all settings, including normal populations [66]. Scores of 0–7 suggest no indication of depression and anxiety, whereas scores ≥8 suggest a potential case of depression/anxiety. Cronbach’s alpha for the depression subscale was α = 0.74 and for the anxiety subscale α = 0.84.

#### 6.3.3. Family Support

We administered the Julkunen Family Support Scale (FSS) [67], a 13-item self-reported scale, for assessing the participants’ perceived family support which they receive from the person(s) they live with. A total score >37 indicates high levels of perceived family support. Cronbach’s alpha for the FSS was α = 0.81.

#### 6.3.4. Assessment of Cognitive Functioning

We employed a range of psychological tasks that cover a wide range of cognitive domains and estimate the overall picture regarding the participants’ cognitive functioning. All participants were tested individually by a trained and experienced psychologist and according to the standard procedures of the test manuals. All administered tasks were standardized for Greek populations, except for one task which was developed for the needs of the present study due to the fact that there was no appropriate test to measure prospective memory; this developed test was based on the study of Eskildsen et al. [16]. Detailed information on the tests can be found in Table A1.

All tests were administered in a constant sequence. Each assessment lasted approximately 60 min and was completed in one session without taking any breaks.

## 7. Statistical Analyses

In order to investigate any statistical relationships between burnout and cognitive functioning, we conducted Pearson correlations with the mean scores of the three MBI-GS subscales, the HADS and FSS total score with the z-scores of each cognitive test. Pearson correlations among MBI-GS, HADS, FSS, cognitive tasks and demographics were also performed. Considering the multiple statistical analyses that were performed and in order to decrease the risk of making Type I errors, the *p* values were adjusted using the Benjamini-Hochberg method [68] (for a review see [69]).

For the statistically significant results between the three MBI-GS subscales and the cognitive tasks, moderation analyses were performed using depression, anxiety and perceived family support as moderators independently in separate moderation models. Additionally, as five different burnout profiles appear to exist, i.e., burnout (high on all three MBI-GS dimensions), engagement (low on all three MBI-GS dimensions), overextended (high on the exhaustion subscale only), disengaged (high on the cynicism subscale only) and ineffective (high on the inefficacy subscale only) [70], we investigated possible differences among the above five burnout profile groups and cognitive functioning by conducting one-way ANOVA multiple comparisons. The burnout profiles were formed according to the cut-off scores provided by Leiter and Maslach [70]. All analyses were performed in SPSS (v.21) and PROCESS (v.3.5) [71].

## 8. Results

### 8.1. Descriptive Characteristics

All variables were assessed for assumptions of normality prior to conducting analyses. All variables were normally distributed, as skewness and kurtosis indices were within the normal range. Details regarding the participants’ characteristics can be found in Table A2.

### 8.2. Correlation Analysis between Demographics, MBI-GS, HADS, FSS and Cognitive Performance

Table A3 depicts the significant correlations observed among the studied variables and the participants’ demographic characteristics.

Concerning the sector which the participants worked in (public or private), private sector employees reported significantly higher exhaustion levels compared to public sector employees [*t*(102) = 2.46, *p* < 0.05] while public sector employees reported higher levels of perceived family support [*t*(74) = 2.75, *p* < 0.05].

### 8.3. Cognitive Performance and Burnout

No statistically significant relationships were observed between exhaustion and performance on the cognitive tests. Cynicism was significantly and negatively correlated with visuospatial abilities, as measured by the first condition of the Taylor Complex Figure Test (TCFT) (*r* = −0.19, *p* < 0.05). Cynicism was also positively associated with participants’ automatic processing skills as it was significantly correlated with the first condition of the Stroop test (Stroop-Word) (*r* = 0.19, *p* < 0.05) (see Table A4). It should be noted that personal efficacy was found to be significantly related with inhibition, as measured by the (third) incongruent condition of the Stroop test (*r* = 0.20, *p* < 0.05). However, after the Benjamini-Hochberg correction this association was indicated as non-significant. No other statistically significant relationships were found.

Concerning the moderation analysis, as the total working hours per week for the second occupation was found to be significantly correlated with the first Stroop condition, we controlled for this variable in the cynicism–Stroop-Word model path. Moderation analysis showed no interaction effects between burnout, depression, anxiety and perceived family support in cognitive performance (all *p*’s > 0.05) (see Table A5).

Continuously, we conducted one-way ANOVA with multiple comparisons to examine any possible differences between the five profile burnout groups on cognitive performance. We categorized our participants according to the five profile burnout groups proposed by Leiter and Maslach [70]. However, as only one participant fitted the criteria for the ineffective profile, we did not include this particular profile in our analysis and categorized the participant’s data as missing. Our results showed that there was a statistically significant difference as determined by univariate ANOVA (*F*(3,98) = 3.931, *p* = 0.01) between the four profile groups in the copy condition on the TCFT. Post hoc analysis using the Tukey test revealed that the participants on the disengaged profile performed significantly worse on the TCFT-copy condition compared to the overextended participants (M = −1.17, SD = 0.35, *p =* 0. 007). No other statistically significant differences were observed.

### 8.4. Burnout, Family Support, Depression and Anxiety

Negative correlations were found between the total FSS score and exhaustion (*r* = −0.24, *p* < 0.05) and cynicism (*r* = −0.30, *p* < 0.01). No significant correlations were found between the total FSS score and personal efficacy (*r* = 0.16, *p* > 0.05). With respect to the relationship between burnout and depression, exhaustion was positively correlated with the total HADS score for both depression (*r* = 0.41, *p* < 0.01) and anxiety (*r* = 0.41, *p* < 0.01). Cynicism was also significantly correlated with the HADS total scores on both scales (*r* = 0.45, *p* < 0.01 for depression; *r* = 0.45, *p* < 0.01 for anxiety). Lastly, personal efficacy was positively correlated with depression (*r* = −0.33, *p* < 0.01) and anxiety (*r* = −0.25, *p* < 0.01). Total FSS score was negatively correlated with both depression (*r* = −0.42, *p* < 0.01) and anxiety (*r* = −0.37, *p* < 0.01) (see Table A6).

### 8.5. Cognitive Performance and Family Support

Perceived family support was associated with long-term visuospatial memory and short-term memory. Specifically, the total FSS score was significantly correlated with participants’ performance on the TCFT-delayed recall trial (*r* = 0.32, *p* < 0.01) and with the forward condition of the Digit Span test (*r* = 0.24, *p* < 0.05), respectively. No other significant relationships were found.

## 9. Discussion

Previous studies have shown that burnout can have negative effects on cognitive functioning [15,17,34,72,73], whereas others have not found such associations [6,18,37,38,39]. Present results do not show robust evidence that burnout is associated with cognitive performance. Specifically, no significant correlations between exhaustion and cognitive performance were found. However, cynicism was related with diminished visuospatial skills. Interestingly, when we examined for any differences in cognitive performance among the five burnout profiles, disengaged participants also showed worse visuospatial skills compared to the other burnout profiles. Considering that the disengaged burnout profile is characterized by employees high only in cynicism, these results suggest that the effects of cynicism on employees’ cognitive functioning might be more negative compared to the other two burnout components. Indeed, cognitive deficits are mainly observed among employees who report high exhaustion feelings [5,74]. Exhaustion has long been considered the core burnout component [70] while exhaustion feelings are the most widely reported from burned out employees [2]. According to the basic conceptual burnout model, exhaustion is considered to develop first and then cynicism follows [21]. However, present results advocate towards the multidimensionality of burnout [75,76,77] and also suggest a different conceptualization of the burnout experience by proposing that cynicism’s effects, at least on cognitive performance, could be stronger than those of exhaustion, a finding in agreement with the observations of studies examining the theoretical background of the burnout experience and which consider cynicism, and not exhaustion, as the core burnout component [21,70].

Another interesting observation was that cynicism was also associated with better performance in the first condition of the Stroop task, indicating better automatic processing skills. This was an unexpected result which may reflect the burnt out employees’ strategies to overcome their difficulties by enhancing their efforts [78,79], or could suggest individual differences in stress experience. According to Williams et al. [44], individuals with better executive functions are less vulnerable to the negative effects of stress while they are more capable of reacting instantaneously when they face a stressor, a process known as stress reactivity, an observation that could also explain the results of Castaneda et al. [39]. Nevertheless, the cross-sectional design of our study does not allow for causality examinations. More longitudinal studies of causality effects are needed in order to shed more light on the exact relationship between burnout and cognitive functioning. To the authors’ knowledge, so far only Feuerhahn et al. [5] have examined for reversed causality effects by showing that executive function deficits are a consequence of exhaustion, and not vice versa.

Although not statistically significant, there was an interesting and positive relationship between personal efficacy and inhibition skills, an observation indicating that, as individuals’ personal efficacy increases, they might be better able to inhibit irrelevant stimuli under conditions of high stress. This result is somewhat in line with the study of Morgan et al. [80] who found that greater perceptions of self-efficacy are related to better executive functions during stressful situations. Positive associations between high levels of personal efficacy and cognitive performance were not unexpected. In fact, even employees with low levels of personal efficacy are able to sustain their job performance as they develop and maintain strategies in order to compensate for their mental fatigue [78,79], such as focusing on the significant tasks and neglecting the less important ones. Nevertheless, perceived levels of work efficacy alone might not act as a protective factor against burnout.

A second goal of our study was to examine whether depression and/or anxiety are inversely associated with cognitive impairment and if they moderate the burnout–cognitive functioning relationship, since the two psychological phenomena have been associated with both burnout [48,49,81,82] and cognitive decline [52,55,56,83,84,85]. However, and in agreement with previous studies [39,83,86,87], we were unable to replicate these results as we failed to find such associations. Moreover, both depression and anxiety did not moderate the burnout–cognitive functioning relationship. It is worth noting that most studies that show cognitive impairments among depressed and anxious individuals mainly pertain to clinical populations [52,84,88] while studies relating to the general population might represent milder depressive and anxiety symptoms. Indeed, in our study most participants showed moderate or no depressive/anxiety feelings. Thus, it is possible that cognitive decline might be an aftermath of major depressive and anxiety disorders or, since these three conditions are frequent in the population, they could develop in tandem by chance [89]. Moreover, individuals with major depressive and anxiety disorders might also seek medication treatment, thus it is possible that the observed cognitive impairment could be mainly a result of medication treatments rather than a symptom of these psychiatric disorders per se. Nevertheless, cross-sectional or longitudinal studies with short time periods might not be able to detect cognitive deficits among depressive and anxious populations. Indicatively, in the study of Ganguli et al. [90], although cross-sectional associations between depression and cognitive impairment were observed, subsequent cognitive decline after a 12-year follow-up was independent of depression, results that indicate the importance of taking into consideration the multifactorial dimension of these conditions when examining their effects.

Some researchers argue that both depression and burnout are the same constructs [49,91,92], whereas others agree that they are differentiated from each other [93,94]. In a recent meta-analysis by Koutsimani et al. [48] it was found that, although depression and burnout appear to share some common characteristics, their association is not strong enough to imply that they are in fact the same construct. The results of this meta-analysis were similar for the burnout–anxiety relationship. In the present study we found that all three MBI-GS subscales were positively (exhaustion and cynicism) and negatively (personal efficacy) correlated with both depression and anxiety. However, the effect sizes among these relationships were medium, supporting the notion that burnout, depression and anxiety, although related, are different constructs.

An additional goal of our study was to verify if the subjective feelings of family support are associated with burnout and cognitive functioning and whether this moderates the burnout-cognitive functioning relationship. Our results showed that high levels of perceived family support were inversely related with exhaustion and cynicism, suggesting the role of family support as a non-work factor than can either protect from, or enhance, burnout onset, while it was also negatively related with both depression and anxiety. Moreover, although the present findings showed that perceived family support was positively related with long-term visuospatial memory, it did not moderate the burnout–cognitive relationship. So far, few studies have examined the effects of family support in both mental health and cognitive performance linking high perceived family support with better cognitive performance [47] and lower burnout levels [46]. Nevertheless, considering various circumstances that require people working from home (e.g., the COVID-19 pandemic), the role of family support in individual health should be examined more extensively.

Lastly, another interesting observation was the existence of a possible, sixth, burnout profile consisting of two dimensions (high on both exhaustion and cynicism), a finding which adds to the observations of Leiter and Maslach [21]. However, the researchers concluded that they could not be certain if this is a potential, sixth, burnout profile or if the disengaged profile needs to be redefined. As, in our study, we located distinct groups of participants who were either high only in cynicism (or exhaustion) or high both in exhaustion and cynicism, our findings support the notion that a sixth burnout profile might exist. Future studies are needed in order to gain a better picture of the characteristics of the burnout profiles.

Our results overall are in accordance with previous studies that did not find any significant relationships between burnout and multiple cognitive domains. This differentiation might be related to the sample variance compared to previous studies, as most of these were conducted with clinical burnout populations. In our study we did not include clinical populations and none of the participants were on sick leave due to burnout symptoms. Additionally, not all studies have examined for other mental disorders (e.g., depression, anxiety), so coexistence of possible cases of other mental disorders could influence results.

## 10. Practical Implications

The findings of the present study indicate the importance of considering all three burnout aspects when examining its effects on employees’ health. Although exhaustion has long been considered the core burnout component which manifests itself prior to cynicism and inefficacy, present results suggest that cynicism could arise first during the early burnout stages, possibly as a coping strategy for alleviating work difficulties. The same reasoning applies when examining cognitive performance. Indeed, most studies that investigate the cognitive abilities of burned out employees focus mainly on the assessment of executive functions [10]. However, present results suggest that other cognitive domains such as visuospatial abilities and automatic processing skills are related to burnout. This, along with the administration of more complex cognitive tasks, is of importance when examining non-clinical burnout populations where the burnout effects are more difficult to be detected. Moreover, burnout can be masked as depression and/or anxiety, leading to false diagnosis and, thus, false treatment. Our findings emphasize the need for thorough examination of individuals who report anxiety and/or depressive feelings. The potential role of one’s perceived levels of support should be taken into consideration by experts when forming intervention programs for individuals with mental health and cognitive disturbances.

## 11. Limitations

One strength of the present study is the broad examination of the cognitive functions we examined as well as the investigation of the role of depression, anxiety and perceived family support in both burnout and cognitive performance. However, our study has several limitations. Firstly, this study is of a cross-sectional design, thus causal relationships cannot be inferred between the associated variables. Future studies should explore these associations through longitudinal designs in order to test for causality, or reciprocal, effects. Secondly, we used snowball sampling as our sampling method. As this is a non-probability method, it is possible that not every person in the population had an equal chance of being selected, so the risk of bias and the risk of error could be higher [95]. Moreover, the sample size was relatively small, consequently making our findings more susceptible to committing Type I errors. Future studies should involve more participants in order to gain a better picture of the studied associations.

Levels of burnout, depression, anxiety and family support were all assessed by self-administered scales. Clinical examination might have provided more accurate results regarding the actual levels of the studied variables. Another limitation could be the cognitive tasks that were administered for examining the participants’ cognitive functions. Since we focused only on non-clinical burnout employees, we are not able to know whether more complex cognitive tasks could detect more subtle cognitive difficulties.

Furthermore, the variability of our sample in terms of demographic characteristics could affect the present results. The participants’ working experience, for instance, ranged from two to 35 years. Employees with fewer years of working experience appear to be more prone to developing burnout [96]. However, in our study we did not find any significant associations between years of working experience and burnout. Moreover, other factors that could affect both burnout and cognitive performance (e.g., daily stress outside the workplace, low social interactions, etc.) that were not examined could also affect our results. The educational level of our sample was also fairly high. Therefore, we are not able to know if the participants’ high educational level has affected the present findings. More heterogenous samples in terms of educational and socioeconomic status could provide different mental health and cognitive patterns.

Lastly, another limitation we faced concerned the assessments’ procedure which was conducted according to the participants’ working schedule. Ideally, when examining individuals’ cognitive functioning, the examination should take place in the morning in order for the individuals to be able to exhibit optimal performance. However, since in our study we included participants who were actively working, depending on their convenience, some of the assessments took place either in the morning before their work or in the early afternoon, during their break, or in their day off. Consistent cognitive assessment schedules for all participants, preferably early in the morning, could provide more accurate results.

## 12. Conclusions

The results of our study add to the existing literature on which factors may lead to burnout and whether burnout is related to cognitive impairment. Moreover, our findings highlight the role of family support in mental and cognitive health, a role of crucial importance when we consider the external factors that often force people to work from home.

## Data Availability

The data presented in this study are available upon reasonable request from the corresponding author.

## Appendix A

**Table A1:** Description of the tasks in the cognitive assessment battery.

Cognitive Task	Description	Cognitive Domain
Taylor Complex Figure Test (TCFT) [97,98]	The TCFT consists of four phases in total; (1) copy; participants are asked to copy the complex figure, (2) immediate recall; participants are asked to draw by memory the complex figure, (3) delayed recall trial; after 30–45 min the participants are asked to draw again by memory the complex figure, and (4) recognition; the participants are asked to recognize parts of the complex figure’s items among disruptive ones. The maximum score for the three first conditions is 36, whereas the maximum score on the fourth condition is 24. The minimum score depends on the age range and the educational level of each participant.	Visuospatial Constructional Abilities & Visuospatial Memory
Short Story [99]	This test consists of two phases; immediate and delayed recall trial. Firstly, a brief story is presented orally twice consecutively and then the participant is asked to recall it and retell it immediately after it was read. After approximately 20 min participants are asked to recall the story again (delayed recall trial). The story is divided in eight parts in total and the scoring depends by the participant’s ability to recall certain parts of the story of each of the eight parts as accurately as possible. Each parts chunk’s maximum score is two resulting to a total maximum score of 32 (combined score of the immediate recall trial) in the first condition and 16 in the second condition. The total score on the delayed recall trial is used for measuring the participant’s performance in episodic memory.	Learning & Episodic Memory
Prospective Memory [16]	Self-made test; at the beginning of the examination the participants are instructed to remember asking the examiner the question: “What time is it?” after the completion of each task. The total score of the test is the total number of questions each participant asked this question after each cognitive task, resulting to a maximum score of 13.	Prospective Memory
Wechsler Adult Intelligence Scale-IV Digit Span (forward, backwards, ascending order) [100]	The participants firstly are asked to repeat ongoing sequences of numbers forward (a task of verbal short-term memory; a part of the working memory system), backwards and in an ascending order. A working memory index score is provided based on the performance in the above three conditions and is transformed in a standardized total score.	Short-term Memory, Verbal Working Memory
Corsi Block-Tapping Span (backwards) [101]	In this test participants are shown an ongoing sequence of blocks and they are asked to recall each sequence backwards.	Visuospatial Working Memory
Trail Making Test (TMT) (part A & B) [102]	Part A: the participants need to connect a sequence of numbers, as quickly as possible.Part B: the participants need to connect a sequence of numbers and letters while alternating between the two. The total time taken to complete each of the two tests is used as the primary performance metric.	Executive functions: visual attention & task switching
Stroop Color-Word Test [103]	This consists of three conditions. Firstly, the participants are required to speedily read a list of color words, then name the color of a list of letters and, lastly, name the color of a list of color words printed in a different color as fast as possible within 45 s.	Executive Functions: Inhibition, Attention, Speed of processing

**Table A2:** Sample Description (*n* = 104).

Variable	*n* (%)
Age (mean, *SD*)	40.40 (10.06)
Range	23–58
Males/Females	24/80 (23.1/76.9)
Education (mean, *SD*)	16.82 (1.39)
Range	12–22
Sector	
Public	62 (59.6)
Private	42 (40.4)
2nd Occupation	22 (21.1)
Years of Working Experience (mean, SD)	15.20 (8.67)
Range	2–35
Family Status	
Cohabitating	8 (7.7)
Married	47 (45.2)
In a relationship	1 (1)
Separated	4 (3.8)
Divorced	3 (2.9)
Single	41 (39.4)
Children	
Yes/No	40 (38.5)/64(61.5)
HADS-Depression ≥ 8 (mean, *SD*)	17 (10.70, 2.49)
Range	8–17
HADS-Anxiety ≥ 8 (mean, *SD*)	39 (10.25, 2.44)
Range	8–18

**Table A3:** Correlations between Demographics, MBI-GS, HADS, FSS and cognitive tasks (*n* = 104).

Variable	Mean	SD	Exh.	CY	HADS-A	FSS	Short Story	Stroop-W	Digit Span-FW	Digit Span-Asc.
Hours/week-main	32.80	11.19	0.29 **	0.23 *	0.20 *	0.37 **	0.24 *	0.00	−0.08	0.17
Hours/week-second	1.80	4.71	0.20 *	0.24 *	−0.00	−0.10	−0.07	−0.26 **	−0.05	−0.00
Hours/week-total	34.60	12.38	0.36 **	0.19 *	0.18	0.06	0.19 *	−0.10	−0.10	0.21 *
Sector	-	-	0.23 *	0.02	−0.07	−0.30 *	0.05	0.13	−0.04	0.09
Family Status	-	-	−0.17	−0.02	0.06	0.23 *	0.00	0.07	0.08	0.08
No of Children	0.83	1.31	−0.09	−0.11	−0.06	−0.13	−0.19 *	−0.05	0.21 *	−0.04

Note: Exh = Exhaustion; CY = Cynicism; HADS-A = Hospital Anxiety and Depression Scale-Anxiety; FSS = Family Support Scale; Short Story = first condition recall; Stroop W = Stroop Word condition; Digit Span FW = Digit Span forward condition; Digit Span-asc. = Digit Span ascending order condition; Hours/week-main = Working hours for the main occupation; Hours/week = Working hours for the second occupation; Hours/week total = Total working hours for the first and second occupation; Sector = Public and Private; Family Status = Single, In a Relationship, Cohabitating, Married, Separated, Divorced. ** *p* ≤ 0.01 level (2-tailed); * *p* ≤ 0.05 level (2-tailed).

**Table A4:** Mean Scores, Standard Deviations (SD) and Correlation Coefficients among the three MBI Scheme 104.

Variables	Mean	SD	1	2	3	4	5	6	7	8	9	10	11	12	13	14	15	16	17	18	19	20
1. E	2.89	1.54	-																			
2. CY	2.08	1.30	**0.42**	-																		
3. PE	5.05	0.96	−0.09	**−0.31**	-																	
4. TCFT-C	33.57	2.74	0.02	−0.19 *	0.17	-																
5. TCFT-IR	25.31	5.22	0.02	−0.16	−0.03	0.09	-															
6. TCFT-DR	24.77	5.04	−0.05	−0.07	0.04	**0.44**	0.13	-														
7. TCFT-Rec.	19.27	2.15	−0.07	−0.09	0.00	0.21 *	0.11	**0.29**	-													
8. Short Story-IR	21.43	5.45	0.00	0.04	0.06	0.23 *	0.00	0.14	−0.03	-												
9. Short Story-DR	11.69	2.92	0.10	−0.01	0.10	**0.37**	−0.02	**0.27**	0.08	**0.74**	-											
10. Prosp.Mem	8.88	3.93	0.02	0.02	0.10	0.05	−0.20 *	0.02	−0.14	0.24 *	0.17	-										
11. STROOP-W	104.08	15.49	−0.02	0.19 *	0.00	−0.10	0.00	−0.01	−0.01	0.02	−0.09	0.03	-									
12. STROOP-C	74.24	10.23	−0.03	0.02	0.07	0.09	−0.08	0.05	0.00	0.01	0.05	0.16	**0.42**	-								
13. STROOP-CW	47.64	9.81	−0.02	−0.03	0.20 *	−0.09	−0.12	0.07	0.06	0.15	0.13	0.07	**0.30**	**0.42**	-							
14. STROOP-Int.	4.47	9.96	−0.00	−0.10	0.19	−0.07	−0.08	0.04	0.03	20 *	0.19*	0.10	−0.11	0.04	**0.77**	-						
15. Digit Span-FW	9.27	2.08	0.00	−0.07	−0.00	−0.00	−0.05	−0.09	0.07	−0.06	0.11	0.00	−0.12	−0.03	0.00	0.06	-					
16. Digit Span-Back.	9.22	2.48	0.05	−0.04	0.07	0.21 *	−0.02	−0.19 *	0.05	0.12	0.15	0.14	0.09	**0.37**	**0.29**	0.25 *	0.22 *	-				
17. Digit Span-Asc.	9.86	2.50	−0.01	−0.09	0.01	**0.27**	0.07	**−0.30**	0.16	0.21 *	**0.25**	0.10	−0.08	0.19 *	0.06	0.14	0.15	**0.41**	-			
18. Digit Span-tot.	28.36	5.98	−0.02	−0.09	0.02	0.22 *	0.02	0.23 *	0.09	0.10	0.18	0.07	−0.12	**0.32**	0.15	0.14	**0.29**	**0.83**	**0.67**	-		
19. Corsi Span- back.	28.43	5.52	−0.11	−0.06	0.01	0.19 *	−0.02	**0.29**	0.16	0.18	0.14	0.14	0.01	0.22 *	**0.32**	**0.31**	−0.01	**0.46**	**0.26**	**0.37**	-	
20. TMT-A	38.68	17.30	0.03	0.04	0.04	−0.01	−0.02	−0.13	−0.18	−0.12	−0.03	−0.04	**−0.27**	−0.13	**−0.27**	−0.17	−0.12	−0.13	−0.01	−0.03	−0.19	-
21. TMT-B	72.02	27.71	0.01	0.16	0.00	−0.18	−0.12	**−0.28**	−0.17	−0.17	−0.24 *	−0.00	−0.15	**−0.31**	**−0.25**	−0.19 *	−0.20 *	−0.37 *	**−0.34**	**−0.40**	**−0.41**	**0.50**

Note. E = Exhaustion, CY = Cynicism, PE = Personal Efficacy, TCFT-C = Taylor Complex Figure Test-Copy trial, TCFT-IR = Taylor Complex Figure Test-Immediate Recall trial, TCFT-DR = Taylor Complex Figure Test-Delayed Recall trial, TCFT-Rec. = Taylor Complex Figure Test-Recognition trial, Short Story-IM = Short Story Immediate Recall trial, Short Story-DR = Short Story Delayed Recall trial, Prosp.Mem. = Prospective Memory, STROOP-W = STROOP Word, STROOP-C = STROOP Color, STROOP-CW = STROOP Color-Word, STROOP-Int. = STROOP Interference, Digit Span-F = Digit Span Forward, Digit Span-B = Digit Span Backwards, Digit Span-Asc. = Digit Span Ascending Order, Digit Span- tot. = Digit Span total score, Corsi Span-back. = Corsi Span backwards, TMT-A = Trail Making Test, Part A, TMT-B = Trail Making Test, Part B.). * *p* ≤ 0.05 level (2-tailed). Bold values denote statistical significance at the *p* < 0.01 level (2-tailed).

**Table A5:** Linear models of predictors of cognitive performance (*n* = 104).

Model Path	*b*	*SE B*	*t*	*p*
Cynicism TCFT-copy				
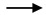
Constant	0.00 [−0.19, 0.21]	0.10	0.07	0.93
Cynicism (centered)	−0.15 [−0.30, −0.00]	0.07	−2.06	0.04 *
Anxiety (centered)	0.00 [−0.04, 0.05]	0.02	0.25	0.79
Depression (centered)	−0.01 [−0.07, 0.05]	0.03	−0.32	0.74
Perceived Family Support (centered)	−0.00 [−0.02, 0.19]	0.01	−0.35	0.72
Cynicism × Anxiety	0.03 [−0.00, 0.07]	0.02	1.51	0.13
Cynicism × Depression	−0.00 [−0.05, 0.05]	0.02	−0.11	0.90
Cynicism × Perceived Family Support	0.00 [−0.01, 0.01]	0.00	0.03	0.96
Cynicism Stroop-W				
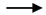
Constant	0.10 [−0.09, 0.30]	0.10	1.00	0.31
Cynicism (centered)	0.14 [−0.00, 0.28]	0.72	1.96	0.051
Anxiety (centered)	0.00 [−0.04, 0.30]	0.02	0.03	0.97
Depression (centered)	0.00 [−0.05, 0.06]	0.03	0.01	0.98
Perceived Family Support (centered)	0.00 [−0.02, 0.02]	0.01	0.00	0.99
Cynicism × Anxiety	0.00 [−0.02, 0.02]	0.12	0.00	0.64
Cynicism × Depression	−0.16 [−0.06, 0.03]	0.26	−0.60	0.54
Cynicism × Perceived Family Suport	−0.00 [−0.02, 0.01]	0.02	−0.59	0.55

Note: TCFT-copy = Taylor Complex Figure Test-copy trial, Stroop-W = Stroop Word, * *p* < 0.05 level (2-tailed).

**Table A6:** Mean Scores, Standard Deviations (SD), Cronbach’s’ α and Correlation Coefficients among the three MBI subscales, HADS and FSS scores (*n* = 104).

Variables	Mean	SD	1	2	3	4	5	6
1. EX	2.89	1.54	(0.90)					
2. CY	2.08	1.30	0.42 **	(.71)				
3. PE	5.05	0.96	−0.09	−0.31 **	(0.84)			
4. HADS-D	5.02	3.24	0.41 **	0.45 **	−0.33 **	(0.74)		
5. HADS-A	6.33	3.82	0.41 **	0.45 **	−0.25 **	0.80 **	(0.84)	
6. FSS	51.40	8.88	−0.24 *	−0.30 **	0.16	−0.42 **	−0.37 **	(0.81)

Note: EX = Exhaustion, CY = Cynicism, PE = Personal Efficacy, HADS-D = Hospital Anxiety and Depression Scale-Depression, HADS-A = Hospital Anxiety and Depression Scale-Anxiety, FSS = Family Support Scale, ** *p* < 0.01 level (2-tailed). * *p* ≤ 0.05 level (2-tailed).
